# Prevalent versus incident progressive supranuclear palsy: An analysis of the frequencies of neuropathological and clinical features at U.S. Alzheimer’s Disease Research Centers indicate a relatively common tauopathy of aging

**DOI:** 10.21203/rs.3.rs-10119056/v1

**Published:** 2026-07-01

**Authors:** Benjamin J Cushing, Ryan K. Shahidehpour, Erin L. Abner, Gregory A. Jicha, Janna M. Neltner, Madeline K. Breig, Kailen L. Teodorescu, Allison M. Neltner, Elif P. Coskun, Gabor G. Kovacs, Peter T. Nelson

**Affiliations:** University of Kentucky; University of Kentucky; University of Kentucky; University of Kentucky; University of Kentucky; University of Kentucky; University of Kentucky; University of Kentucky; University of Kentucky; University of Kentucky; University of Kentucky

**Keywords:** Scanscope, epidemiology, FTLD-Tau, MAPT, 4R tauopathy, AGD

## Abstract

Progressive supranuclear palsy (PSP) is a neurodegenerative disease diagnosed according to its histopathologic pattern of tau proteinopathy (“tauopathy”). It is increasingly appreciated that PSP is heterogeneous in both clinical and pathological presentations. However, the prevalence of PSP subtypes, in comparison to other tauopathies, remain incompletely characterized. Here we analyzed NACC Neuropathology Data Set data aggregated from 37 U.S. Alzheimer’s Disease Research Centers (ADRCs). Clinical and gold-standard neuropathologic features of autopsied participants were compared, stratifying on cognitive status at recruitment into the study. The final sample comprised 6994 individuals who were followed approximately annually for 4.0 years on average before autopsy. Among those with dementia at recruitment (n=4309), 2.9% had autopsy-confirmed corticobasal degeneration (CBD), 2.6% Pick’s disease, and 4.6% PSP. By contrast, among those recruited while cognitively normal (n=1452), 0.7% had CBD, 0.1% Pick’s disease, and, remarkably, 3.3% were diagnosed with PSP pathology. The relatively high frequency of PSP pathology detected among individuals recruited while cognitively normal suggests there is a subtype of PSP that is unexpectedly common in the broader population. In comparing between incident (recruited normal) and prevalent (recruited with dementia) autopsy-confirmed PSP, those with incident PSP died older (89.6 years versus 76.2 years on average). Furthermore, incident PSP pathology cases were less likely to manifest stereotypical PSP clinical features, but more likely to have parkinsonism, compared to prevalent PSP pathology cases. In a convenience sample of autopsy-confirmed PSP from the University of Kentucky ADRC (n=23), digital pathology analyses using HALO software and AI-based analytic modules revealed that PSP tau pathology was more severe in prevalent PSP, but in the putamen, incident cases had a higher proportion of tufted astrocytes and lower proportion of NFTs. In summary, incident PSP pathology is a relatively common tauopathy in older ADRC participants, often differing clinically and pathologically from prevalent PSP.

## Introduction

Progressive Supranuclear Palsy (PSP) is an adult-onset neurodegenerative disease with a heterogeneous spectrum of clinical and pathological features, and the epidemiology of PSP (as defined by the gold-standard of pathological disease) remains unresolved.[17] Steele, Richardson, & Olszewski originally described nine cases with vertical gaze ophthalmoplegia, pseudobulbar palsy, dysarthria, and dystonia, now known as classic PSP, or PSP with Richardson syndrome (PSP-RS).[21, 55] However, pure PSP-RS constitutes as little as 24% of clinical PSP.[45] Additional clinical phenotypes were subsequently described, including PSP with predominant parkinsonism (PSP-P) and at least seven other subtypes.[21, 44] Refinements of PSP clinical criteria are ongoing and improvement of clinicopathological correlation for PSP remains a topic of interest. [21][2, 43, 53, 54]

As with other neurodegenerative diseases, autopsy is required for definitive diagnosis of PSP as well as determination of its pathological severity. Early PSP studies described neurofibrillary tangles (NFTs, composed of microtubule-associated protein tau), particularly in the brainstem and basal ganglia.[55] Subsequently, tau-immunoreactive tufted astrocytes (TAs) were identified as a more pathognomonic feature.[63] Further work has characterized PSP, along with corticobasal degeneration (CBD), argyrophilic grain disease (AGD), and glial globular tauopathy (GGT), as 4-repeat tauopathies.[48] In 1994, a “preliminary” set of histopathological criteria for PSP was published -- the National Institutes of Neurological Disease and Stroke (NINDS) criteria,[20] which required a semiquantitative evaluation of NFT and tau neuropil thread density in several basal ganglia and brainstem regions. However, the NINDS criteria suffered from poor inter-observer reliability, particularly in “atypical” and “combined” PSP cases. [32] Subsequent research determined in more granular detail the range of pathologies in PSP and the anatomic regions of interest affected at different stages.[29] The more recent (2022) Rainwater Charitable Foundation criteria for autopsy diagnosis of PSP incorporated TAs in either peri-Rolandic cortices or putamen, along with NFTs or pretangles in at least two of globus pallidus, subthalamic nucleus, and substantia nigra.[47]

The epidemiology of PSP is an important dimension of its public health impact, but gathering population-level data on PSP is challenged by several practical barriers and potential pitfalls. Due to its clinical heterogeneity and variable overlap with more common conditions such as Alzheimer’s disease (AD) and Parkinson disease, PSP can be difficult to diagnose clinically. [56] The uncertainty of its epidemiology is compounded by the fact that only a small proportion of the population undergoes autopsy, required for diagnosis, with autopsy rates declining in recent decades.[36] Estimates of the prevalence of PSP (mostly operationalized according to stereotypical clinical manifestations) vary widely, from 1/100,000 to 18/100,000, as do estimates of incidence, from 0.16/100,000 person-years to 2.6/100,000 person-years.[12, 33] Other sources indicated an unexpectedly high PSP pathologic frequency in older individuals (see Ref [14]).

The varying results of prior PSP epidemiology studies are partly a reflection of differences in study design. Recruitment into many high-quality academic studies comes from specialty neurology clinics, leading to recruitment bias, with participant samples that are often enriched for early-onset, stereotypical and pathologically “pure” cases.[16] Such clinic-based studies provide invaluable insights into disease biology but may skew perceptions about the clinicopathological range of the disease phenotype and ultimately obscure the epidemiology. The results of large-scale epidemiologic studies based on clinical outcomes (e.g., Ref [12]) can also reflect biases in terms of the clinicopathological correlation. To avoid concerns about the recruitment biases of specialty clinics and studies depending only on clinical data, community-based autopsy cohorts recruit neurologically normal participants and follow them longitudinally for years (often for decades); many of the participants eventually coming to autopsy with comprehensive neuropathological examination. These studies have biases of their own, including a tendency to study older (> 85 years at death) autopsied participants. Nonetheless, data from research centers deploying this study design provide a scientifically useful opportunity to assess neuropathological phenomena with broader generalizability to older populations.[16]

Two widely used terms in the study of disease at the population level are prevalence and incidence. A condition’s prevalence indicates the number of existing cases in a defined population at a point in time, whereas incidence reflects the number of new cases of disease in a defined population.[40] Given the low autopsy rates in most populations, the prevalence and incidence of PSP are difficult to estimate with confidence. However, when following a cohort of research volunteers longitudinally to autopsy and defining the presence and severity of the disease by the pathologic observations, one can apply related terms: there are prevalent cases, defined as individuals who were neurologically impaired at the time of recruitment, and incident cases, defined as individuals who became impaired during the course of longitudinal follow-up while on study. Parenthetically, the connotation of the term “incident pathology” is quite different from that of the term “incidental pathology”, which implies that the person died without any neurological impairment.[15]

Clinical and pathological indices can vary substantially when comparing between prevalent and incident cases of Alzheimer’s disease and related dementias (ADRD). We previously found that ADRD-focused autopsy cohorts that recruited a relatively high proportion of neurologically normal individuals reported autopsy outcomes that were systematically different from the cohorts that recruited more participants with dementia.[16] Specifically, ADRD autopsy cohorts that specialize in prevalent cases reported a higher frequency of “pure” (single) pathologies and younger-onset conditions, with a considerable enrichment of rare diseases that led to reported prevalence rates that were not generalizable to broader populations. By contrast, cohorts that had more incident disease comprised participants who tended to be older and more female, were enriched for “mixed pathologies,” with few or no frontotemporal dementia (FTD) cases, and these cohorts provided a more generalizable approximation for the prevalence of the pathology in human populations.[16]

Digital pathology and AI-based image analyses provide an opportunity to extend neuropathologic assessment beyond traditional semi-quantitative scoring by enabling reproducible, high-throughput quantification and classification of pathology. When paired with automated detection and classification algorithms, whole-slide imaging allows pathology to be assessed according to continuous and spatially resolved measures. While conventional semi-quantitative assessments remain essential for diagnosis, they may not capture the differences in lesion density, and thus digital pathology and AI-based quantification may help to better define pathological heterogeneity. Digital pathology and/or AI methodology have previously been utilized to differentiate PSP from other tauopathies and to assess PSP severity.[11, 25–27, 42, 49].

In the current study, we sought to compare clinical and pathologic features of incident and prevalent PSP and other tauopathies, using data from US National Institute on Aging-funded Alzheimer’s Disease Research Centers (ADRCs), complemented by digital pathologic assessments in a select subsample. Most of the analyzed data were obtained from the National Alzheimer’s Coordinating Center Neuropathology (NACC NP) Data Set.[7] NACC aggregates both longitudinal clinical and pathologic information from dozens of ADRCs, allowing correlation between participants’ findings at autopsy with the course of their antemortem symptoms. We hypothesized that a comparison between incident and prevalent PSP could elucidate differences in terms of demographics, clinical features, and pathologic parameters at autopsy.

## Materials and methods

### NACC NP Data Set

Data were obtained on January 13, 2026 from the NACC NP Data Set, referent to the December 2025 data freeze.[8, 9] Participants enrolled in ADRC cohorts were assessed using the standardized Uniform Data Set (UDS) forms at their local ADRC during annual clinic visits. The UDS includes demographic information, degree of cognitive impairment, general health information such as current medications, diagnoses, and social history, and neurological signs and symptoms such as amnestic dementia, PSP-associated symptoms, and parkinsonism.[7] For participants who underwent autopsy, a NACC NP data form was submitted from the relevant ADRC.[1] This form included detailed gross and microscopic assessment of pathologic features including AD neuropathologic change (ADNC), Lewy body neuropathologic changes, and frontotemporal lobar degeneration (FTLD) subtypes including PSP.

For the pathological diagnosis of PSP, each ADRC utilized the common NACC NP data form which has been in use since 2014.[1] This data form enables one to diagnose “FTLD-tau (PSP)” which can be selected as “Yes,” “No,” “Not assessed,” or “Missing/unknown.” Further, the data form indicated that “[Pathologic] Evaluation should follow published guidelines. For details of specific diagnoses and a classification diagram of FTLD subtypes, see the Coding Guidebook for the NACC Neuropathology Data Form.” In the Coding Guidebook, Dickson [13] was cited for guidance in regard to PSP pathologic diagnosis.

We reviewed the NACC NP Data Set focusing on data from participants for whom autopsy information was available. Clinical and pathologic features were compared across groups stratifying by initial cognitive status: normal, mild cognitive impairment (MCI), and dementia. Participants not classifiable into one of these three categories of cognitive status (most of which were “Impaired, not MCI”) were excluded from analysis. We repeated these comparisons on the subset of cases meeting pathologic criteria for PSP, allowing a comparison of clinical and pathologic features among those with incident and prevalent PSP. Demographic and clinical information assessed included sex, race, age at initial visit, age at death, number of total ADRC visits, and percentage having dementia at final ADRC visit. Neuropathologic features assessed include the three measures of ADNC: Thal Aβ phase,[57] Braak NFT stage,[10] and Consortium to Establish a Registry for Alzheimer’s Disease (CERAD) score for cortical neuritic amyloid plaque density,[35], as well as the diagnoses of tau pathologies PSP, CBD, Pick’s disease, argyrophilic grains, “tangle dominant disease”, or other tauopathy. As the latter three conditions did not have their presence or absence assessed in all participants, proportions were based on the subset of participants for whom the presence or absence of disease was specifically evaluated and documented. For participants with confirmed PSP pathology, we examined the percentage who had been clinically diagnosed with PSP, as well as the presence or absence of symptoms consistent with PSP: eye movement changes, dysarthria, axial rigidity, gait disorder, and apraxia of speech. For examination of these particular symptoms of PSP, proportions were based on the subset of participants for whom these symptoms were specifically evaluated and documented.

#### Statistical Analyses of NACC NP Data

Null hypothesis significance testing was focused on comparisons of participants who entered follow-up with either normal cognition or dementia. Participants who entered with a diagnosis of MCI contributed contextual summary data (**Supplemental Tables 1–6**), but were not included in the statistical analyses. Characteristics that were operationalized as categories were compared with chi-squared or Fisher’s exact text as appropriate. Characteristics that were operationalized as continuous variables or counts were compared using t-tests, using Student’s or Sattherwaite’s variance estimator as appropriate. Significance was set at p < 0.05.

#### Tissue Preparation and Immunohistochemistry in a UK-ADRC subsample, for digital pathology

Work flow for the digital pathology studies is depicted in **Supplementary Fig. 1**. Immunohistochemical staining and subsequent analysis were performed across three regions routinely sampled in autopsies at the University of Kentucky Alzheimer’s Disease Research Center (UK-ADRC): dorsolateral prefrontal cortex (corresponding to Brodmann area 9); the putamen at approximately the level of the anterior commissure; and, the midbrain at the level that also includes the red nuclei. These regions were selected because they show pTau pathology in PSP and were collected routinely during neuropathologic autopsy evaluation.

Immunohistochemistry of pTau was carried out on 8 μm sections prepared from formalin fixed paraffin-embedded human autopsy brain tissue. Sections were stained using an antibody directed against pTau (PHF-1 clone, 1:1000, courtesy of the late Dr. Peter Davies).[18, 37, 38, 52] Slides were deparaffinized in xylene and then rehydrated through a graded ethanol series of 100%, 95%, and 70% ethanol before being transferred to distilled water. Antigen retrieval was performed using Dako Target Retrieval Solution (low pH, TRS Lo, pH 6, Cat. #GV805), heated to 95°C for 6 minutes, followed by a 10-minute cooling period at room temperature. Slides were then rinsed in running distilled water for 10 minutes and equilibrated in 1× TBS on a shaker for 5 minutes. To block endogenous peroxidase activity, sections were incubated for 30 minutes in a solution containing 20% methanol and 3% hydrogen peroxide. After rinsing in distilled water and washing in 1× TBS for 5 minutes, tissue sections were blocked for 1 hour at room temperature in 5% non-fat dry milk prepared in 1× TBS. Primary antibody incubation was performed overnight at 4°C in a humidified chamber. The following day, slides were washed twice in 1× TBS for 5 minutes each and then incubated for 1 hour at room temperature with biotinylated horse anti-mouse secondary antibody (Vector Laboratories, #BA-2000, 1:250). After two additional 1× TBS washes, sections were incubated for 1 hour with the ABC Peroxidase, (VECTASTAIN^®^ Elite ABC Peroxidase kit, Vector Laboratories, #PK-6100). Immunoreactivity was visualized using DAB chromogen solution (DAKO, #K3468), applied dropwise until sufficient signal development was achieved. Sections were counterstained with Harris Hematoxylin (#HH-160). All slides were allowed to dry for 48 hours prior to imaging. Following staining, each slide was digitized at 40× magnification using a Leica/Aperio AT-2 whole-slide scanner.

#### Analysis using HALO AI Software

To accurately quantify and phenotype pTau pathology in the PSP cases, a high-throughput histopathology quantification pipeline was developed using the HALO image analysis platform (version 4.1.6, Indica Labs, Albuquerque, New Mexico, USA). This workflow integrated four classifier modules into a single automated pipeline, allowing for streamlined preprocessing and improved tissue classification. The digital pipeline used previously stained and digitized whole slide images (WSIs) and began by removing glass/background and meningeal tissue. The tissue detection classifier was tuned using a resolution setting of 2.0 μm/pixel and gated to only detect objects with a minimum size of 5000 μm^2^. Training for the tissue detection classifier was conducted on > 500 objects and 600,000 training iterations achieving an entropy score of < 0.01. Next, a quality control classifier eliminated tissue folds, tears, areas of poor focus, and extraneous debris from usable tissue. The quality control classifier was similarly tuned using 2.0 μm/pixel resolution and set to detect objects > 100 μm^2^. This classifier was also trained on > 500 objects and completed 400,000 training iterations until its entropy value was < 0.01. Finally, the grey/white matter segmentation classifier was used to distinguish cortical regions and generate distinct annotation layers between grey and white matter to be used for regional analysis. This final classifier was tuned using 7.00 μm/pixel resolution to ignore small variations in grey/white matter staining and only detected objects > 5000 μm2. This classifier was also trained using > 500 objects and underwent 750,000 training iterations that continued after reaching an entropy value of < 0.01. Annotated neocortical regions were separated into a training set composed of 80% of annotated regions, a validation set comprised of 10% of annotated regions, and a test set containing 10% of annotated regions. Classifier validation achieved a precision score of 0.969, a recall score of 0.961 and an F1 score of 0.965.

Following tissue dissection, a Nuclei Segmentation classifier (HALO AI) was used to detect PHF-1 immunoreactive structures, regardless of their pathological phenotype. This classifier was tuned using a 0.5 um/pixel resolution and the minimum object threshold was set at 220 um^2^. This detection/segmentation classifier trained on > 750,000 training iterations reaching a cross-entropy value of < 0.1. Using this nuclei segmentation classifier, an object phenotyper classifier was also set up to delineate between NFTs, tufted astrocytes and coiled bodies.[24, 42] Similar to the nuclei segmentation classifier, this classifier was set to a resolution of .5 um/pixel and underwent > 150,000 training iterations reaching a cross-entropy score of < 0.002. Data outputs from this classifier were reported in number cells/mm^2^ as well as percent of each cell type compared to the total population of cells detected per section. Statistical analyses for digital pathological evaluations were conducted in GraphPad Prism version 10.6.1. Unpaired Welch’s t test (two tailed, without Bonferroni correction) were to assess statistical differences between pathology types in incident and prevalent PSP. Comparisons were considered statistically significant when p < 0.05.

## Results

After exclusion criteria were applied, the NACC NP Data Set sample consisted of 6845 included participants with neuropathological data, of whom 1452 were cognitively normal, 1084 had MCI, and 4309 had dementia at initial ADRC visit (Table 1). The study sample derived from 37 different ADRCs and the median number of included participants per ADRC was 156.5. Note that only 26.9% of those recruited while cognitively normal were documented to have developed dementia before death. In the subgroup analysis of participants for whom detailed information about Thal Aβ phase and overall ADNC data were available, there were 3818 participants in total, of whom 939 were cognitively normal, 710 had MCI, and 2169 had dementia at initial visit (Table 2). In the subgroup analysis of participants for whom neuropathologic information was available regarding argyrophilic grains, tangle dominant disease, and FTLD-tau pathology or other tauopathy, there were 3772 participants in total, of whom 929 were cognitively normal, 695 had MCI, and 2148 had dementia at initial visit (Table 3). For the subgroup of participants with PSP pathology, there were 297 participants in total, of whom 48 were cognitively normal, 57 had MCI, and 192 had dementia at initial visit (Table 4). For the subgroup of participants with confirmed PSP and for whom detailed information about Thal Aβ phase and overall ADNC were available, there were 197 participants, of whom 41 were cognitively normal, 41 had MCI, and 115 had dementia at initial visit. Finally, for the subgroup of participants with confirmed PSP who had detailed clinical information about the presence or absence of their specific PSP symptoms, there were 73 participants in total, of whom 22 were cognitively normal, 17 had mild cognitive impairment, and 34 had dementia at initial visit (Tables 5, 6). For analogous tables that include a column depicting the numbers of participants with initial diagnoses of MCI, please see **Supplemental Tables 1–6**. For the most part, the results for individuals recruited with MCI were intermediate between the findings of participants recruited with normal cognitive status and those recruited with dementia.

There were statistically significant differences between the group of participants who were cognitively normal at initial visit in comparison with those who were diagnosed with dementia at initial visit (Table 1). Persons recruited while cognitively normal were more likely to be female and had older ages at initial visit and at death (P < 0.01). There also were differences in ADNC severity by initial cognitive status (Table 2). Only 16.0% of participants who were initially cognitively normal were found to have high level ADNC, compared with 43.4% of those with MCI and 56.6% of those with dementia at initial visit (P < 0.01). Similarly, only 18.0% of those who were initially cognitively normal were found to have Thal Aβ phase 5, compared with 36.5% of those recruited with MCI and 49.5% of those recruited with dementia (P < 0.01), and 19.8% of those who were initially cognitively normal had frequent neuritic amyloid plaques, compared to 37.0% of those with MCI and 54.4% of those with dementia initially (P < 0.01). Tau pathology (operationalized according to Braak NFT staging) also differed significantly, with both stages 0 and VI most common in those with dementia initially (P < 0.01).

The frequency of PSP pathology in participants initially cognitively normal was 3.3%, compared to 4.5% in those initially with dementia (P = 0.058; Table 3). By contrast, CBD (0.7% versus 3.3%) and Pick’s disease (0.1% versus 3.0%) were significantly less common in those initially normal than those initially with dementia (P < 0.01; Table 3). Other tauopathies showed different patterns: specifically, reported argyrophilic grains (12.2% versus 6.0%) and tangle dominant disease (6.4% versus 1.1%) were more common in participants initially cognitively normal than in those initially recruited with dementia (P < 0.01; Table 3).

Examination of the subset of participants with confirmed PSP pathology revealed no significant differences between those recruited cognitively normal and those recruited with dementia regarding rate of overall high level ADNC, Thal Aβ phase 5, or frequent neuritic amyloid plaques (Table 4). However, there was a significant difference in the distribution of Braak NFT stages between these groups (P < 0.01; Table 4). Participants with PSP pathology who were recruited while cognitively normal were less likely than those recruited with dementia to have been diagnosed clinically with PSP (8.3% versus 48.4%, P < 0.01; Table 5). By contrast, the initially normal participants with eventual autopsy-proven PSP were more likely to have been diagnosed clinically with Parkinson disease (12.5% versus 3.7%, P < 0.05; Table 5). Analyses of specific clinical symptoms among participants with eventual PSP pathology showed that all of the PSP-associated symptoms in the UDS -- eye movement changes (P < 0.01), dysarthria (P < 0.01), axial rigidity (P < 0.01), gait disorder (P < 0.01), and apraxia of speech (P < 0.05) -- were more common among those recruited with dementia than in those recruited cognitively normal (Table 6).

### AI-based detection and phenotyping of PSP- associated pTau pathology

**Figure 1:**
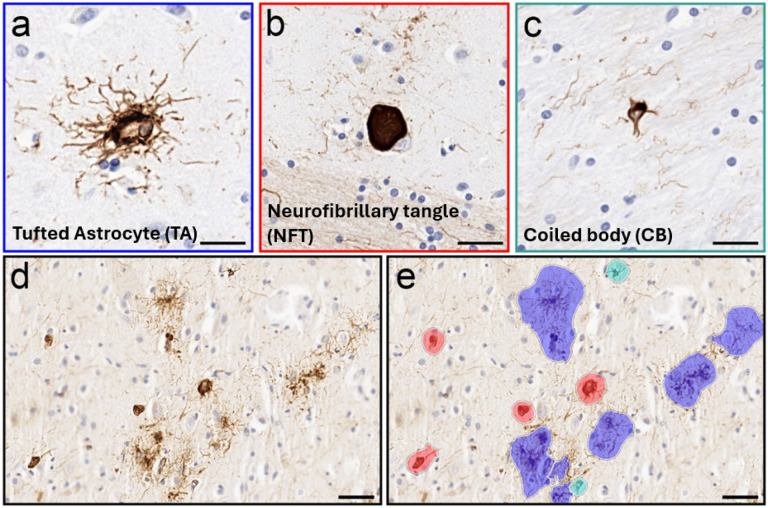
PSP pathology and automated detection and phenotyping of pTau immunoreactive lesions. Representative examples pTau pathology phenotypes and automated detection results. High-magnification examples of pTau-positive morphologies (a-c), including tufted astrocytes (a; TAs; blue), neurofibrillary tangles (b; NFTs; red) and coiled bodies (c; CBs; teal). Panel d shows an unannotated field containing multiple pTau-positive lesions with the corresponding automated detection overlay, with color-coded segmentations identifying lesion classes across the tissue field (e). Scale bars = 50mm (a-c) and 100mm (d-e).

Digital pathology was utilized on a convenience sample of pathologically-confirmed PSP cases from the UK-ADRC. Select demographic, genetic, and co-pathology data from each of these cases are presented in **Supplemental Table 7**. WSIs of pTau immunostained sections were analyzed using an AI-based digital pathology pipeline designed to identify and classify PSP-associated pathology. The classifiers detected and phenotyped three classes of pTau-positive pathology: CBs, NFTs, and TAs. Representative photomicrographs are shown in Fig. 1a–c. The nuclei segmentation/object phenotyper classifier identified pTau-positive structures throughout the tissue section and assigned classification phenotypes, allowing both total pathologic burden and class–specific densities to be quantified within each region of interest (Fig. 1d–e). This approach enabled systematic assessment of PSP pathology as both an overall quantitative burden and as a phenotypically resolved measure of pTau pathology.

**Figure 2:**
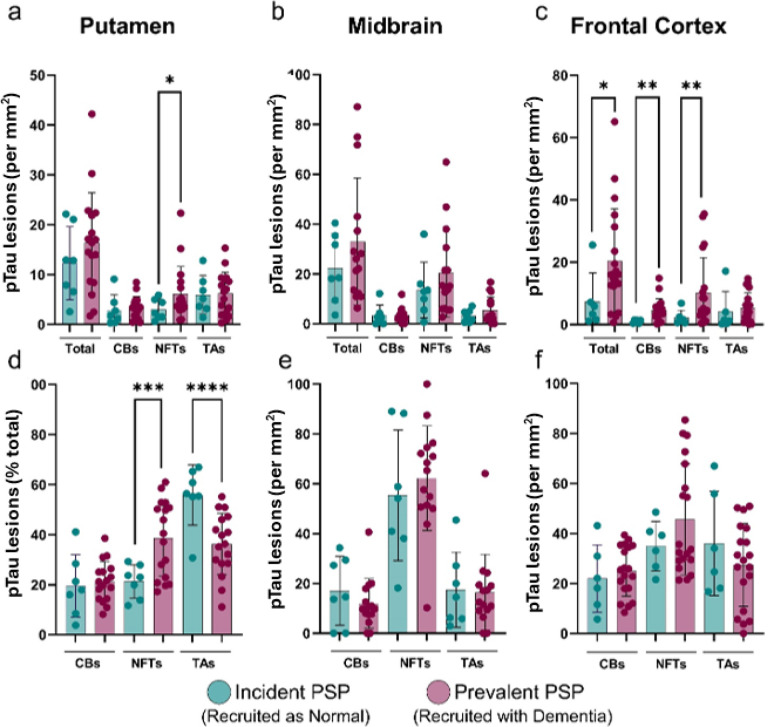
Incident and prevalent PSP cases differ in regional tau lesion density and lesion subtype composition. Quantification of pTau-positive cellular lesions in incident PSP and prevalent PSP across selected brain regions and lesion classes. Incident PSP cases are shown in teal and prevalent PSP cases in magenta. Bars represent group means with error bars indicating standard deviation, individual points represent individual cases. Regional densities of total pTau-positive cells, coiled bodies (CBs), neurofibrillary tangles (NFTs), and tufted astrocytes (TAs) are shown for the putamen (a), midbrain (b), and frontal cortex (c). In the frontal cortex, prevalent PSP showed increased total pTau-positive lesion density, accompanied by higher CB and NFT densities (c). Lesion subtype compositions, expressed as the percentage of total pTau-positive cellular pathology, are represented in panels (d-f), illustrating the relative contribution of CBs, NFTs, and TAs. These analyses showed greater NFT burden in prevalent PSP and greater TA burden in incident PSP within the putamen (d). Brackets indicate between-group comparisons; * = P < 0.05,* = P < 0.01,*** = P < 0.001, **** = P < 0.0001.

AI-based quantification was used to compare the density and cellular composition of pTau pathology within the putamen, midbrain, and frontal cortex between cases of incident and prevalent PSP (Fig. 2). Across regions, prevalent PSP cases showed higher and more variable pTau densities than incident PSP cases. In the putamen, total pTau-positive lesion density was variable in both groups, with substantial overlap between incident and prevalent PSP cases (Fig. 2a). While prevalent cases tended to show higher overall pathology density, the clearest phenotype-specific difference was observed in NFT density, which was significantly increased in prevalent PSP cases compared to incident PSP cases (P = 0.026). CBs and TA densities showed overlapping distributions between groups, suggesting that the distinction between incident and prevalent cases in the putamen was driven primarily by neuronal pTau pathology rather than by a uniform increase across all lesion types. In the midbrain, total pTau density was high in both incident and prevalent PSP cases, and several prevalent cases showed marked elevations in total lesion density (Fig. 2b). However, group differences were not statistically significant for total lesion density or for individual lesion phenotypes. NFTs represented the dominant lesion type in both groups, while CBs and TAs were present at lower densities.

The most pronounced differences between incident and prevalent PSP cases were observed in the frontal cortex (Fig. 2c). Prevalent PSP cases had significantly higher total pTau-positive lesion density than incident PSP cases. Phenotype-specific analysis showed that this overall increase was driven by greater densities of CBs and NFTs in prevalent PSP. TA density showed similar (although not statistically significant) trends between groups. Separate renderings of the digital pathology results are charted in **Supplemental Fig. 2**, to indicate the pTau+ lesion densities and pTau+ lesion subtypes as an overall percent of total.

When lesion phenotypes were expressed as a proportion of the total detected pTau-positive lesional population, prevalent cases showed a significantly greater relative contribution of NFTs, whereas incident cases showed a higher proportional contribution of TAs (P = 0.0001 and P = 0.0008, respectively) (Fig. 2d). Similarly, proportional analysis showed that NFTs accounted for the largest fraction of the detected pTau-positive population in the midbrain for both incident and prevalent cases (Fig. 2e). The overlap between groups may reflect the early and consistent involvement of midbrain structures in PSP, such that even incident cases can show substantial pathology in this region. Furthermore, in the frontal cortex, NFTs accounted for the largest fraction of pTau pathology in prevalent PSP, although the relative contribution of each lesion phenotype varied across cases (Fig. 2f). This suggests that cortical progression in prevalent PSP may involve both increased total burden and a shift in lesion composition toward neuronal and oligodendroglial pTau pathology. Overall, cases of prevalent PSP were associated with greater cortical involvement and increased neuronal pTau pathology in both the putamen and frontal cortex, while cases of incident PSP cases showed lower lesion burden but relatively higher TAs in putamen.

**Figure 3:**
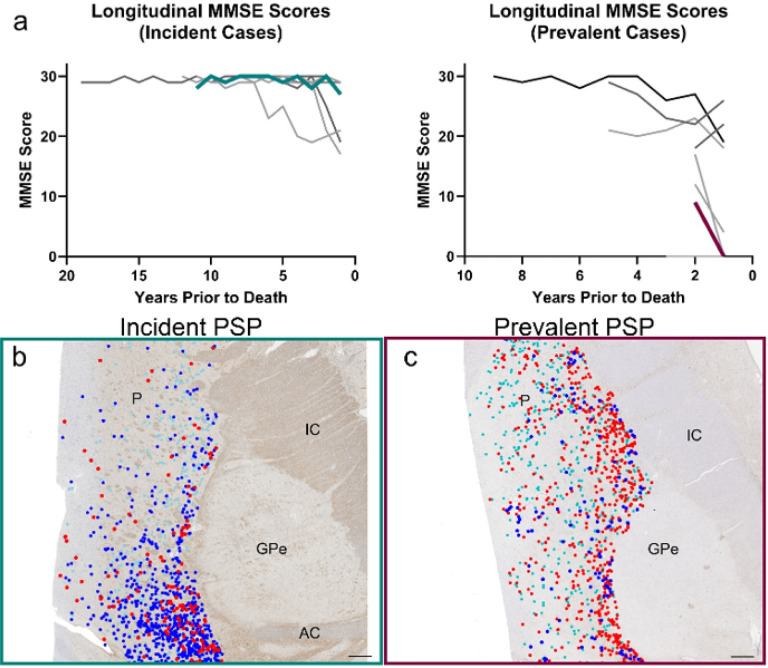
Clinical and neuropathological comparison of incident and prevalent PSP cases from the UK-ADRC. This figure shows the longitudinal cognitive trajectories and spatial distributions of PSP-related pTau pathology in incident and prevalent PSP cases. See**Supplemental Table 7** for detailed information on each case. Longitudinal MMSE scores were plotted as years prior to death for incident PSP cases and prevalent PSP cases (a). Individuals’ results are shown as lines, with color-coded traces corresponding to the representative tissue maps in panels b and c. These show digitized tissue sections from representative incident PSP (b) and prevalent (c) PSP cases, overlaid with automated detections of pTau pathologic lesion subtypes. Colored markers denote detected NFTs (red), TAs (blue), and CBs (teal). Abbreviations: P = putamen, IC= internal capsule, GPe = external segment of globus pallidus, AC = anterior commissure. Scale bars represent 1mm.

Longitudinal MMSE test results for incident (turquoise) and prevalent PSP cases (maroon) are shown in Fig. 3a. Incident cases generally maintained high MMSE scores with a generally longer disease course, and with most scores remaining in the upper range until the years immediately preceding death.

Representative spatial maps of AI-detected pTau lesions illustrate the pathologic features of incident and prevalent PSP cases in the putamen (Fig. 3b–c). In the incident cases, detected pathology were largely comprised of TAs, in contrast, the prevalent PSP cases showed a more extensive and spatially widespread distribution of NFTs.

## Discussion

We compared incident and prevalent disease cases of a subset of tauopathies, including PSP, CBD, Pick’s disease, and ADNC. We found substantial differences between the initially unimpaired (incident disease) and those with dementia at recruitment (prevalent disease). In comparison to other subtypes of FTLD-Tau, PSP showed a high frequency of incident disease, and incident PSP cases were relatively older. Other tauopathies, including argyrophilic grains and tangle dominant disease, were also more common in cases recruited while neurologically normal. By contrast, CBD and Pick’s disease were substantially less frequent among incident disease cases than prevalent disease. Furthermore, there were differences (comparing incident versus prevalent disease cases) in the severity of ADNC (Table 2).

Changes in both clinical and pathologic diagnostic criteria of PSP, while necessary for providing as accurate of a diagnosis as possible, are an inherent limitation of studies such as ours which rely on data collected over decades. In a broader sense, as clinical and pathological criteria for PSP evolve, our understanding of PSP epidemiology may change accordingly. In 2017, the Movement Disorder Society developed new clinical diagnostic criteria, with acknowledgement of specific symptoms corresponding to specific subtypes, to replace the previous 1996 NINDS-SPSP criteria which were highly specific for PSP-RS but lacked sensitivity for variant subtypes.[21, 32]

Estimates of disease epidemiology based on autopsy cohort data may also be influenced by selection bias. While cohorts focusing on recruiting patients with dementia are essential for studying rare disease phenotypes, cohorts which are more representative of broader populations allow for more generalizability of findings, and assessing disease-associated longitudinal trajectories.[16] Because NACC aggregates data from autopsy cohorts at many different research centers with different recruitment methodologies, analysis of NACC data may reflect the selection biases at each center. However, this also makes the NACC data set a useful source of information to compare incident and prevalent disease because both are represented in among the data. Another limitation to generalizability is the demographic makeup of the NACC data set: ADRC cohorts tend to recruit more White participants, more female participants, more participants with relatively extensive formal education, and more participants of high socioeconomic status than are found in the overall population; all of these factors may affect the analytic readouts.[6, 31, 34, 60, 65]

In the present study, we found that PSP pathology occurred in 3.3% of the initially normal participants who came to autopsy, and 4.3% of those with dementia initially (Table 3). Both the detected incident and prevalent PSP frequencies are considerably higher than the published population-level epidemiological data would suggest.[33] Subgroup analysis of participants with confirmed PSP pathology revealed that, of 48 incident cases, only 4 (8.3%) received the clinical diagnosis of PSP before death. By contrast, of 192 prevalent PSP cases, 93 (48.4%) had the clinical diagnosis of PSP (Table 5). The incident PSP cases were over threefold more likely to have been diagnosed with parkinsonism than the prevalent PSP cases (Table 5). Further investigation revealed that the incident cases were significantly less likely to express each the PSP-specific symptoms evaluated in the NACC data set: eye movement changes, dysarthria, gait disorder, and apraxia of speech (Table 6). These findings raise concerns that most PSP cases are not being recognized in the clinical context, particularly in the general population where detailed, serial neurological evaluation is not commonplace. It also suggests that PSP with older-age onset (operationalized as incident cases in the current study) may have different symptomatology and different pathologic findings. Multiple recent autopsy studies have reached similar conclusions that PSP, particularly as defined by the Rainwater criteria, may be much more common than previously thought, with a majority of cases not being diagnosed as PSP during life and many patients not appearing to have dementia.[14, 30, 39, 46, 58, 64]

We note that PSP is generally categorized as a subset of FTLD-tau. This classification may be worthy of reconsidering. Applying the nosology of FTD/FTLD for PSP is potentially misleading in that the predominant pathologies of PSP are not distinctively enriched in either frontal or temporal cerebral lobe, and the signal clinical features of FTD (disinhibition and/or aphasia and/or motoneuron disease) are not particularly associated with PSP pathology. By contrast, although peri-Rolandic tauopathy is common in PSP, the clinical symptoms associated with PSP are largely related to subcortical pathology. Moreover, according to prior work, other non-frontal, non-temporal neocortical brain regions, such as the supramarginal gyrus of the parietal lobe, are important regions involved in PSP-related cognitive impairment.[51]

The use of AI-based digital pathology analysis enhances neuropathological evaluation by providing quantitative, region-specific, and phenotype-resolved measures of PSP-associated pTau pathology. This is particularly valuable in diseases such as PSP, where diagnostic pathology comprises multiple lesion phenotypes with distinct cellular and regional distributions. In the present analysis, automated whole-slide assessment enabled separate measurement of total pTau-positive lesion density and the densities of CBs, NFTs, and TAs across the putamen, oculomotor nucleus within the midbrain, and frontal cortex. The resulting data showed differences between incident and prevalent PSP. Within the putamen, prevalent PSP was characterized principally by increased NFT density, whereas total pTau-positive lesion density, CB density, and TA density showed overlapping distributions. In the midbrain, both groups showed substantial pTau pathology without significant differences in total or subtype-specific lesion densities. However, analysis of the frontal cortex showed the clearest separation between groups, with prevalent PSP demonstrating increased total pTau-positive lesion density driven by higher CB and NFT densities. Thus, the digital pathology results suggest that prevalent PSP is associated less with a uniform increase in all lesion types and more with greater neuronal and oligodendroglial pTau pathology. Further investigation of the proportion of each pathological phenotype demonstrates the utility of AI-assisted lesion phenotyping in PSP. In the putamen, prevalent PSP showed a greater relative contribution of NFTs, whereas incident PSP showed a greater relative contribution of TAs, indicating a shift in the cellular composition of the pTau-positive lesion population (Figs. 2, 3). In contrast, proportional lesion composition in the midbrain and frontal cortex showed substantial inter-case variability without significant group-level differences.

Prior studies using semiquantitative measures have also reported variation in regional tau burden in PSP that broadly were compatible with the current study.[28, 41, 50] More specifically, Badihian et al. found that younger age at death was associated with greater tau burden in subcortical regions in PSP cases [4], and Josephs et al. reported that PSP disease duration was inversely associated with oligodendroglial tau burden.[23] Williams et al., Guasp et al., and Jellinger previously described differences between PSP-RS and PSP-P that also are simpatico with our results.[19, 22, 61, 62] Other prior researchers have also used the NACC data set to study PSP.[3, 5, 59] Distinguishing features of the current study include our large overall cohort with a bias toward older participants, rather than earlier-onset PSP-RS cases, and a focus on pathology-verified cases, while stratifying on the basis of incident vs prevalent cases and combining these analyses with digital pathology. Using this approach, we found that most PSP cases were neither PSP-RS nor PSP-P according to clinical findings, whereas stratification of prevalent versus incident cases can be a useful method to highlight different subtypes of disease.

Several important limitations warrant consideration in interpreting these findings. The NACC database does not specify the exact method of PSP diagnostic workup at autopsy, and in that data set PSP pathological diagnosis is treated as a dichotomous (yes/no) variable. As such, ADRC neuropathologists do not systematically capture neuropathological staging of PSP according to the six-stage framework proposed in 2020, which defines sequential involvement from early subcortical neuronal tau accumulation in the pallido-nigro-luysian axis.[29] It hence is not possible to formally assign disease stages to individual cases, and the observed differences in pTau burden between incident and prevalent groups may also reflect differences in pathological stage. Specifically, incident cases may represent earlier pathological stages, characterized by predominantly subcortical and brainstem involvement with a relatively greater astroglial tau component in the striatum. Prevalent cases, by contrast, may more often represent advanced stages marked by frontal and neocortical neuronal and oligodendroglial pTau accumulation. Furthermore, some small neurons with tangles might be interpreted as coiled bodies by the AI-based classifier; however, the proportion of astroglial compared to neuronal/oligodendroglial pTau would be still different in incident versus prevalent disease. Future studies integrating formal neuropathological staging with AI-based quantification will be necessary to disentangle the contributions of disease stage, clinical recognition, and cellular tau phenotypes.

In conclusion, our findings suggest that incident PSP is relatively common—this pathology is possibly far more frequent among the older population than the classic PSP-RS clinical phenotype. NACC-contributory, ADRC-affiliated dementia clinics presumably were the source of many of the prevalent PSP cases, which tended to have the PSP-RS phenotype, whereas research participants with incident PSP were more likely to have parkinsonian symptom phenotype. Further, incident and prevalent PSP differed in both the overall pTau burden and phenotypical composition of pTau pathology. Using AI-based methods of detection and phenotyping, prevalent cases showed greater pTau burden across sampled regions, with the most pronounced differences in the frontal cortex, where increased total pTau density was driven largely by higher densities of CBs and NFTs. Prevalent cases demonstrated a greater contribution of neuronal pTau pathology, while incident cases showed a relatively greater astrocytic (TA) component, and these same cases had maintained higher cognitive scores over time. Thus, clinical recognition and cognitive decline in PSP may relate not simply to the presence of pTau pathology, but to its regional spread and cellular phenotypes.

## Supplementary Material

Supplementary Files

This is a list of supplementary files associated with this preprint. Click to download.

- SupplementalFiles.docx

## Figures and Tables

**Table 1:** Demographics and neuropathology of included participants, stratified by cognitive status at initial visit

Variable	Initial visit diagnosed as cognitively normal	Initial visit diagnosed as having dementia	Overall	P-value: initial Normal vs initial Dementia
**Sample size (n)**	**1452**	**4309**	**5761**	N/A
% Female	59	43.5	47.4	<0.0001
% White	94.6	93.4	93.7	0.11
Age at initial visit (years) [SD]	80.5 [9.0]	72.4 [11.1]	74.4 [11.2]	<0.0001
Age at death (years) [SD]	88.0 [9.1]	76.7 [11.3]	76.7 [11.9]	<0.0001
# total visits (avg[SD])	6.2 [3.5]	3.2 [2.3]	4.0 [2.9]	<0.0001
% with initial visit only	8.3	28.8	23.6	<0.0001
% dementia at last visit	26.9	99.6	81.2	<0.0001
Median # of participants per ADRC	24	88.5	123	<0.0001

**Table 2:** Select Alzheimer’s disease neuropathologic changes (ADNC), stratified by cognitive status at initial visit

Variable	Initial visit diagnosed as cognitively normal	Initial visit diagnosed as having dementia	Overall	P-value: initial Normal vs initial Dementia
**Sample size (n)**	**1452**	**4309**	**5761**	N/A
% with high level ADNC^a^	16.0	56.6	44.3	<0.0001
% with Thal Aβ phase 5^a^	18.0	49.5	40.0	<0.0001
% with frequent neuritic plaques	19.8	54.4	45.6	<0.0001
% with Braak NFT stage 0	3.3	8.0	6.8	<0.0001
% with Braak NFT stage I	11.4	6.8	8.0	
% with Braak NFT stage II	19.9	6.7	10.0	
% with Braak NFT stage III	19.8	6.4	9.7	
% with Braak NFT stage IV	24.0	9.0	12.8	
% with Braak NFT stage V	12.3	18.1	16.2	
% with Braak NFT stage VI	7.6	42.4	33.7	

a Effective sample sizes: normal = 939, dementia = 2169

**Table 3:** Frequencies of various tau pathologies (PSP, CBD, Pick’s, argyrophilic grains, tangle dominant disease, and FTLD-Tau in general), stratified by cognitive status at initial visit

Variable	Initial visit diagnosed as cognitively normal	Initial visit diagnosed as having dementia	Overall	P-value: initially Normal vs Dementia
**Sample size (n)**	**1452**	**4309**	**5761**	N/A
% with PSP	3.3	4.5	4.2	0.058
% with CBD	0.7	3.3	2.7	<0.0001
% with Pick’s disease	0.1	3.0	2.3	<0.0001
**Subsample size (n)** ^a^	**929**	**2148**	**3077**	N/A
% with argyrophilic grains (NPFTDT5)^a^	12.2	6.0	7.8	<0.0001
% with tangle dominant disease (NPFTDT9)^a^	6.4	1.1	2.7	<0.0001
% with FTLD-tau related pathologies or other tauopathies (NPFTDTAU)^a^	26.2	23.0	24.0	0.058

a Effective sample sizes for analyses of NPFTDT5, NPFTDT9, and NPFTDTAU

**Table 4:** Select parameters and Alzheimer’s disease neuropathologic changes (ADNC), stratified by cognitive status at initial visit, among participants with eventual autopsy-confirmed PSP

Variable	Initial visit diagnosed as cognitively normal	Initial visit diagnosed as having dementia	Overall	P-value: initially Normal vs Dementia
**Sample size (n)**	**48**	**192**	**240**	N/A
% Female	52.1	38	40.8	0.076
% White	97.9	95.8	96.3	0.69
Age at initial visit (years) [SD]	79.3 [8.7]	72.8 [8.1]	74.1 [8.6]	<0.0001
Age at death (years) [SD]	89.6 [7.9]	76.2 [8.6]	78.9 [10.0]	<0.0001
# total visits (avg [SD])	8.0 [3.8]	2.8 [1.9]	3.8 [3.2]	<0.0001
% with initial visit only	2.1	33.9	27.5	<0.0001
% dementia at last visit	45.8	98.4	87.9	<0.0001
% with high level ADNC^a^	5.3	11.6	9.6	0.36
% with Thal Aβ phase 5^a^	12.2	11.3	11.5	0.88
% with frequent neuritic amyloid plaques	20.8	14.6	15.8	0.31
% with Braak NFT stage 0	0.0	15.6	12.5	<0.0001
% with Braak NFT stage I	0.0	16.1	12.9	
% with Braak NFT stage II	12.5	15.6	12.5	
% with Braak NFT stage III	20.8	10.9	12.9	
% with Braak NFT stage IV	31.3	9.9	14.2	
% with Braak NFT stage V	4.2	7.8	7.1	
% with Braak NFT stage VI	8.3	9.4	9.2	

a effective sample sizes: normal = 41, dementia = 115

**Table 5:** Select clinical features of participants with autopsy-confirmed PSP, stratified by cognitive status at initial visit

Variable	Initial visit diagnosed as cognitively normal	Initial visit diagnosed as having dementia	Overall	P-value: initially Normal vs Dementia
Persons with pathologic dx of PSP, n	**48**	**192**	**240**	N/A
Persons with pathologic dx of PSP, also with clinical dx of PSP, % (FINAL VISIT)	8.3	48.4	40.4	<0.0001
Persons with pathologic dx of PSP, also with clinical dx of Parkinson dis., % (FINAL VISIT)	12.5	3.7	5.4	0.026

**Table 6:** Clinical features among participants with clinical data available related to PSP-associated symptoms, stratified by cognitive status at initial visit

Variable	Initial visit diagnosed as cognitively normal	Initial visit diagnosed as having dementia	Overall	P-value: initially Normal vs Dementia
Persons with detailed clinical readouts of PSP, n	**435**	**481**	**916**	N/A
Persons with detailed clinical readouts of PSP, also with pathologic dx of PSP, n	**22**	**34**	**56**	N/A
Persons with detailed clinical readouts of PSP, also with pathologic dx of PSP, %	5.1	7.1	6.1	0.2
Persons with detailed clinical readouts of PSP, also with pathologic dx of PSP: % with eye movement changes consistent with PSP (FINAL VISIT)	4.5	47.1	30.4	0.0007
Persons with detailed clinical readouts of PSP, also with pathologic dx of PSP:% with dysarthria consistent with PSP (FINAL VISIT)	4.5	47.1	30.4	0.0007
Persons with detailed clinical readouts of PSP, also with pathologic dx of PSP:% with axial rigidity consistent with PSP (FINAL VISIT)	4.5	47.1	30.4	0.0007
Persons with detailed clinical readouts of PSP, also with pathologic dx of PSP:% with gait disorder consistent with PSP (FINAL VISIT)	4.5	47.1	30.4	0.0007
Persons with detailed clinical readouts of PSP, also with pathologic dx of PSP:% with apraxia of speech (FINAL VISIT)	4.5	29.4	19.6	0.036

## Data Availability

All data used in this article will be made available to the research community in a reasonable timeframe. More specifically, all specific HALO modules used for digital pathology (parameters and coding) assessments will be shared immediately on publication.

## Abbreviations

AD: Alzheimer’s disease
ADNC: Alzheimer’s disease neuropathic change
ADRC: Alzheimer’s disease Research Center
ADRD: Alzheimer’s disease and related dementias
AGD: argyrophilic grain disease
APOE: apolipoprotein E
BA: Brodmann area
CB: Coiled body
CBD: Corticobasal degeneration
CERAD: Consortium to Establish a Registry for Alzheimer’s Disease
FTD: Frontotemporal dementia
FTLD: Frontotemporal lobar degeneration
FTLD-tau: FTLD with tau inclusions
LATE-NC: Limbic predominant age-related TDP-43 encephalopathy neuropathologic change
MCI: Mild cognitive impairment
MMSE: Mini Mental State Exam
NACC: National Alzheimer’s Coordinating Center
NFT: Neurofibrillary tangle
NINDS: National Institutes of Neurologic Disease
PART: Primary age-related tauopathy
PSP: Progressive supranuclear palsy
pTau: hyperphosphorylated tau protein
ROI: region of interest
TA: Tufted astrocyte
UDS: Uniform Data Set
UK-ADRC: University of Kentucky Alzheimer’s Disease Research Center
WSI: whole slide image
